# Micro-Injection Moulding In-Line Quality Assurance Based on Product and Process Fingerprints

**DOI:** 10.3390/mi9060293

**Published:** 2018-06-11

**Authors:** Federico Baruffi, Matteo Calaon, Guido Tosello

**Affiliations:** Department of Mechanical Engineering, Technical University of Denmark, Produktionstorvet Building 427A, 2800 Kgs. Lyngby, Denmark; mcal@mek.dtu.dk (M.C.); guto@mek.dtu.dk (G.T.)

**Keywords:** micro-injection moulding, quality assurance, process monitoring, micro metrology

## Abstract

Micro-injection moulding (μIM) is a replication-based process enabling the cost-effective production of complex and net-shaped miniaturized plastic components. The micro-scaled size of such parts poses great challenges in assessing their dimensional quality and often leads to time-consuming and unprofitable off-line measurement procedures. In this work, the authors proposed a novel method to verify the quality of a three-dimensional micro moulded component (nominal volume equal to 0.07 mm^3^) based on the combination of optical micro metrology and injection moulding process monitoring. The most significant dimensional features of the micro part were measured using a focus variation microscope. Their dependency on the variation of µIM process parameters was studied with a Design of Experiments (DoE) statistical approach. A correlation study allowed the identification of the product fingerprint, i.e., the dimensional characteristic that was most linked to the overall part quality and critical for product functionality. Injection pressure and velocity curves were recorded during each moulding cycle to identify the process fingerprint, i.e., the most sensitive and quality-related process indicator. The results of the study showed that the dimensional quality of the micro component could be effectively controlled in-line by combining the two fingerprints, thus opening the door for future µIM in-line process optimization and quality assessment.

## 1. Introduction

Microsystems are among the main drivers of the technological evolution introduced by the information age. Consequently, the demand for small components whose dimensions are in the micrometric and nanometric scale has largely increased in numerous engineering fields over the recent decades [1]. In this context, micro components made of thermoplastic polymers became more and more widespread due to the reduced weight, high chemical resistance, low production cost and ease of fabrication, even in complex shapes. Most of these products are nowadays produced by micro-injection moulding (µIM). This process was ideated as miniaturized version of the conventional injection moulding process (IM), with the aim of combining its high productivity with micro manufacturing capabilities [2]. If, on one hand, the two technologies share the same process cycle phases (plasticization, injection, packing, cooling and ejection), on the other, they have substantial differences [3]. Firstly, dedicated µIM machines having separate elements for plasticization and injection have to be used if tolerances in the micrometre range are the production target [4]. Since the positive outcome of any replication process strictly depends on the dimensional accuracy of the master, new micro tooling technologies were developed to manufacture moulds with features having micrometric dimensions [5]. Another discrepancy between IM and µIM relates to the filling of the cavity, which becomes much more challenging in the micro-scale. In fact, as the injected melt volume is extremely small, the surface-to-volume ratio increases, and thus a very fast solidification occurs, hindering the complete filling of the cavity [6]. Therefore, in order to favour the replication capability of the process, high levels of injection speed, melt temperature and mould temperature are typically required [7,8,9]. Since the levels of these parameters are superiorly limited by the occurrence of polymer degradation, the process window of µIM becomes narrower than that of IM, making process optimization a fundamental step for manufacturing products that comply with design specifications. However, µIM optimization is made difficult by the fact that, when the geometrical characteristics of the components are the response variables, time-consuming experimental investigations based on off-line, high-accuracy dimensional measurements are necessary to tune the process. In fact, numerous features have to be assessed at the same time by means of state-of-the-art measurement systems. Typically, optical instruments are preferable for this task [10,11,12] because of their contactless nature and sub-micrometric resolution. A possible solution to this issue is the identification of a single measurable characteristic of the part that is strongly statistically correlated to the other measurands and therefore to the overall product quality. By assessing only this dimensional outcome, which can be referred to as the “product fingerprint”, the conformance to the specifications of all functional tolerances could be ensured. The product fingerprint must be also sensitive to the variation of process settings in order to work as an optimization tool for µIM; a change of process parameters has to be reflected in a variation of the fingerprint value if an effective control over the process has to be performed.

Another typical quality assurance issue of µIM and other moulding processes is that their extremely high throughput rates do not allow for measurement of all the produced parts with three-dimensional instruments [13], and therefore the production is verified by measuring a few random components extracted from the manufactured batch. This approach, which is favoured by the industry for its cost efficiency, is unsuitable for micro productions, where micrometric tolerances require an extremely high process repeatability and therefore a total quality assurance approach. A solution to this problem is the use of µIM process monitoring. The application of process monitoring to IM has been often reported in literature. Most studies [14,15,16] agree upon the centrality of cavity pressure as the process variable that best summarizes the evolution of the moulding cycle and determines the final part shrinkage. If, on one hand, the usage of a pressure sensor inside the cavity is generally without risks for a conventional moulded part, on the other, the size of micro plastic components is comparable or even larger than that of typical transducers, therefore impeding their use without drastically changing the cavity shape and the part design [17]. A possible solution to this problem is monitoring the pressure provided by the injection screw or plunger (the so-called hydraulic pressure in IM). In fact, this quantity is always stored in the machine data for each moulding cycle and can be extracted for analysis without the need to use any further sensor. The main drawback of this approach is the substantial difference between cavity and hydraulic pressures due to pressure losses generated within the nozzle and feed system and to the high compressibility of polymer melts. Therefore, the pressure measured at the plunger might not be representative of how the polymer melt is behaving inside the cavity.

In the field of µIM process monitoring, Griffiths et al. examined the effect of the main µIM process parameters on cavity pressure [18], demoulding forces [19], melt temperature [20], and air evacuation from the cavity [21]. They concluded, in all cases, that the selection of different process settings had a relevant impact on the recorded conditions, demonstrating that µIM can be successfully controlled by monitoring suitable process variables. However, the authors did not investigate the correlation between those variables and the dimensional quality of the produced micro components. If a strong correlation between the part dimensions and a monitored process indicator, called the “process fingerprint”, is established, the quality assurance can be carried out in-line by only controlling its value. The process fingerprint must be also influenced by the variation of the main µIM process parameters in order to be used as an optimization tool. By finally correlating the product and process fingerprints, an experimental approach to carry out an in-line control of the main part features can be established and implemented, reducing the quality control time and simultaneously enhancing its robustness.

The present paper introduces a study aimed at the identification of the product and process fingerprints for the µIM process of a three-dimensional micro component for medical applications. The most important geometrical part features were selected and the effects of the variation of process parameters were studied by applying a statistical Design of Experiments (DoE) approach. A correlation study was then performed to identify the feature being mostly correlated with the others, i.e., the product fingerprint. In-line process monitoring was also applied during the moulding experiments; injection pressure and injection velocity curves were analysed with respect to process variations and the best process fingerprint candidate was identified. Finally, the correlation between the process and product fingerprints was investigated to establish an effective in-line quality assessment procedure for the micro part.

## 2. Materials and Methods

### 2.1. Case Study

**Figure 1:**
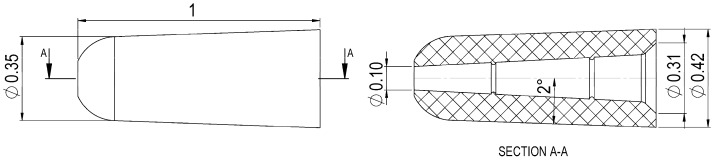
Geometry and nominal dimensions in mm of the micro component.

The produced part was a polyoxymethylene (POM) micro part used in medical applications. Figure 1 shows its main dimensions and three-dimensional shape. The two structures on the internal surface and the 2° tape of the outer conical surface were designed to facilitate the ejection of the part from the mould. Since its nominal volume equalled 0.07 mm^3^ (equivalent to a nominal mass of 0.1 mg) and the dimensional tolerances of the diameters were specified as ±10 µm, the part belonged to the category of micro moulded products according to the standard definitions [22]. Table 1 reports some examples of the volumes of micro moulded components reported in literature. The extremely small size of the part made the use of any in-cavity sensor inapplicable.

### 2.2. Mould Design

**Figure 2:**
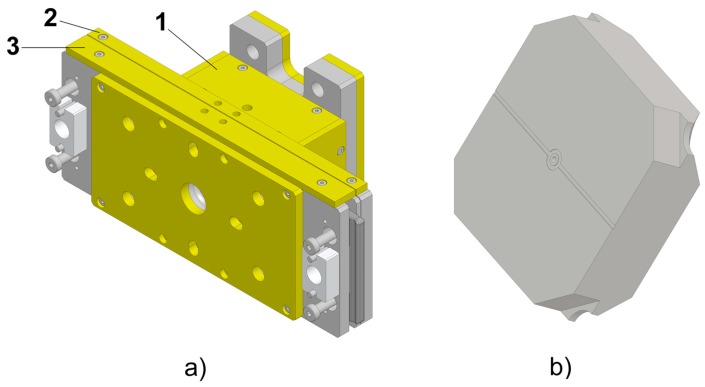
(**a**) 3D model of the mould: 1, 2, and 3 indicate ejection, middle, and injection plates respectively. (**b**) Replaceable insert with venting channel machined on the opposite side of the injection point.

**Figure 3:**
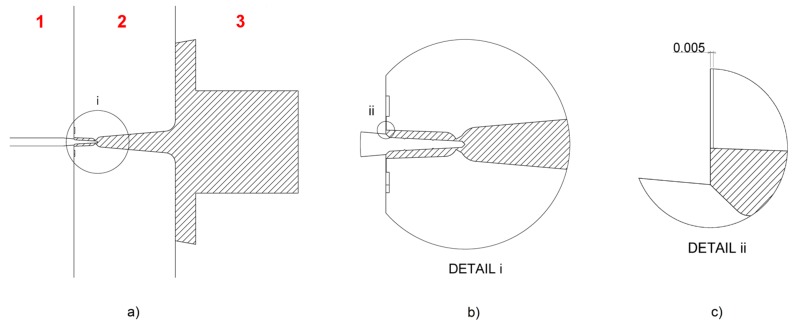
Mould design. (**a**) Mould cross-section showing the part and feed system location (hatched) with respect to the three mould plates (numbered in red). (**b**) Detail of the moulded component and ring gate. (**c**) Detail of the venting channel machined on the insert at the end of the flow path (nominal thickness in mm).

The micro components were moulded using a replaceable insert made of tool steel mounted in a three-plate mould (see Figure 2a). Such a mould configuration had the main advantage of enabling the automatic separation of the component from the feed system. This feature becomes particularly useful when small parts are produced since it avoids a manual gate separation that inevitably introduces variability in the production process. The structured hole was created by replicating a micro pin protruding from the movable plate of the mould. The insert cavity and the pin were both machined using micro electro-discharge-machining (µEDM). The feed system consisted of a cylindrical sprue with a 5 mm diameter, a conical runner, and a ring gate that had a nominal thickness of 25 µm and was axially symmetric with respect to the part. The ejection was carried out by means of a vacuum gripper mounted on a robot arm, thus ensuring a fully automated µIM process. Figure 3 illustrates the mould design and the location of the part within the mould frame.

Preliminary experiments highlighted the need of a venting channel to achieve complete filling of the cavity. The cause of this was identified as the presence of entrapped air in the cavity, and therefore a circular 5 µm deep venting channel was machined by µEDM on the back of the insert (see Figure 2b and Figure 3c) to obtain consistent part filling. If, on one hand, such modification allowed the issue related to the unfilling to be solved, on the other, it generated a flash defect around the largest outer diameter of the component since the polymer melt was allowed to flow inside the venting channel.

### 2.3. Experimental Details

**Figure 4:**
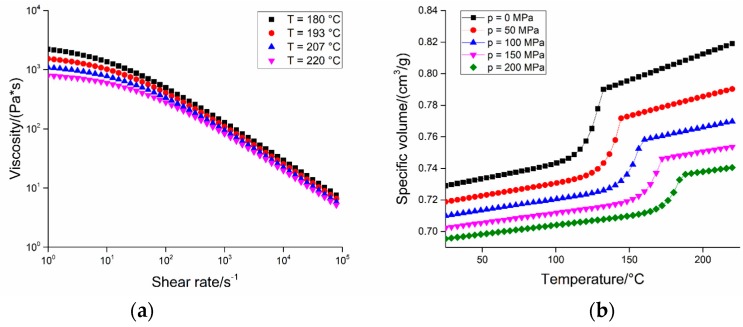
Viscosity plot at different temperatures (**a**) and *pvT* data at different pressures (**b**) for the polyoxymethylene (POM) grade.

Micro moulding experiments were carried out using a state-of-the-art Wittmann Battenfeld MicroPower 15 µIM machine (Kottingbrunn, Austria, maximum injection velocity: 750 mm/s, maximum clamping force: 150 kN). This machine has a plasticization screw and a separate injection plunger; the first has a diameter of 14 mm and the function of melting and homogenizing the polymer, while the second has a diameter of 5 mm and drives the melt inside the cavity with the desired speed and pressure. The material used was an unfilled POM (Hostaform^®^ C 27021, Celanese, Irving, TX, USA). This grade was selected for its peculiar properties, namely its low friction coefficient, good mechanical properties, and extremely low melt viscosity. Table 2 reports the main characteristics of the material and Figure 4 shows its viscosity and *pvT* data.

**Figure 5:**
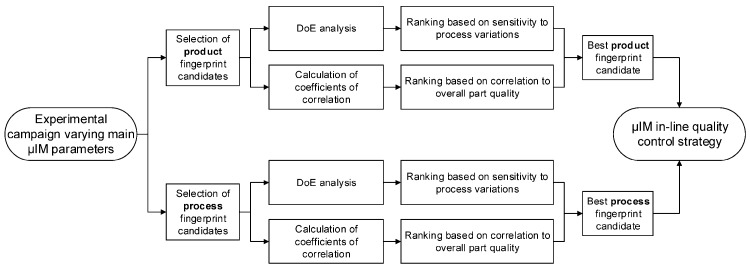
Flowchart representing the identification method for product and process fingerprints. The procedure starts with the selection of product and process fingerprint candidates. Design of Experiments (DoE) and correlation analyses allow for the determination of the best candidates and for the definition of the micro-injection moulding (µIM) in-line control strategy.

In order to identify the best product and process fingerprints, an experimental campaign was carried out by varying the main µIM process parameters. Such a study was necessary since, as already anticipated, both the product and process fingerprints must be sensitive enough to process settings variations in order to function as optimization tools. Moreover, the data gathered through the experimental campaign were also used to determine the indicator that had the highest level of correlation to part quality, that is, the other characteristic needed to carry out an effective in-line quality control based on process monitoring. Figure 5 shows the flowchart representing the general procedure used for determining the best product and process fingerprints.

A Design of Experiments (DoE) approach was adopted. Four process parameters, namely holding pressure, injection speed, mould temperature and melt temperature were varied according to a two-level full factorial design. Such variables were selected since they are widely reported in literature to be the ones having the largest influence on the replication capability as well as on the level of shrinkage of moulded products [32,33,34,35,36]. The designed experimental plan allowed for the evaluation of the effects of the single process parameters and of their interactions with the maximum resolution. The levels of the investigated variables were set according to preliminary moulding experiments and the material manufacturer’s recommendations. Table 3 shows the details of the DoE experimental plan. It can be observed that the holding pressure values were set at a relatively low value for µIM; this was adopted in order to avoid an excessive flash formation that would have disabled the part functionality. For each combination of process parameters, the first ten produced parts were discarded and the following five were kept for evaluation, thus making five DoE replicates available for the following analysis.

### 2.4. Measurement Strategy and Uncertainty Evaluation

**Figure 6:**
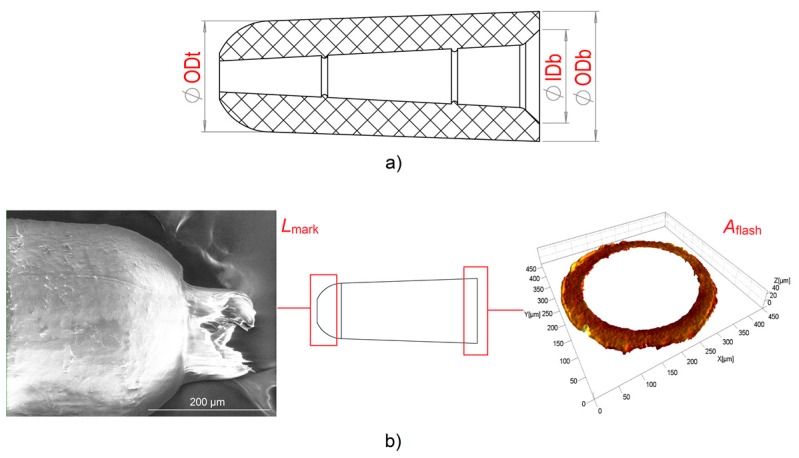
Product fingerprint candidates. (**a**) The three diameters outer top diameter (ODt), inner bottom diameter (IDb) and outer bottom diameter (ODb). (**b**) The two part defects and respective size indicators: the area of the flash (*A*_flash_) and the length of the gate mark (*L*_mark_).

Five dimensional features of the part were selected as product fingerprint candidates and assessed for each of the 80 produced parts. Three of them were functional geometries of the component. These are shown in Figure 6a and are the outer top diameter (ODt), outer bottom diameter (ODb), and inner bottom diameter (IDb). The other two were related to the main defects affecting the part quality: the flash and the gate mark. The flash, as already anticipated, was generated by the presence of the venting channel. The gate mark was caused by the detachment of the feed system from the part by means of the displacement of the middle plate and appeared as an unwanted prolongation in correspondence with the gate area (see Figure 6b). Both defects had to be minimized in order to ensure the part functionality, and therefore their size was a straightforward optimization response and an ideal product fingerprint candidate. The flash and the gate mark were quantified by means of dimensional indicators: the area of the flash, *A*_flash_, was used to characterize the flash size, while the length of the gate mark, *L*_mark_, was chosen as indicator of the gate mark size (see Figure 6b). Therefore, *A*_flash_ and *L*_mark_ both increase when the two defects become larger.

The five product fingerprint candidates were measured on each moulded part with a 3D state-of-the-art focus variation microscope (InfiniteFocus, Alicona Imaging GmbH, Raaba, Austria) using a 20× magnification objective lens. Table 4 reports the main instrument characteristics in the used acquisition mode.

**Figure 7:**
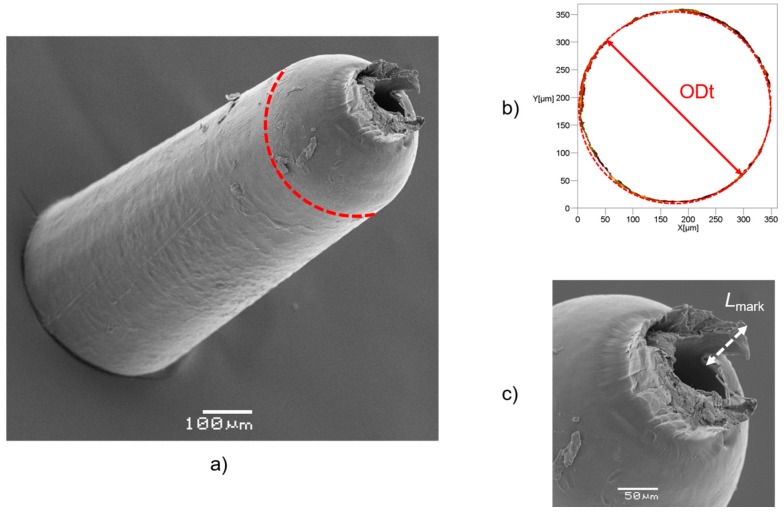
(**a**) SEM image of a moulded part; outer top diameter (ODt) is indicated in red. (**b**) Measurement of ODt. (**c**) Definition of *L*_mark_.

**Figure 8:**
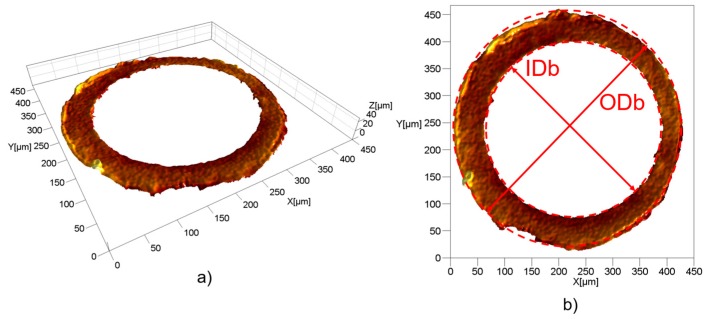
(**a**) 3D acquisition of the bottom of a moulded part. (**b**) Measurement of inner bottom diameter (IDb) and outer bottom diameter (ODb).

In order to capture the five measurands, two acquisitions were carried out for each moulded sample. The top side of the part, i.e., its left side in Figure 1, was acquired and then the measurands, ODt and *L*_mark_ were extrapolated. The bottom of the part, i.e., its right side in Figure 1, was instead acquired to measure ODb, IDb and *A*_flash_. The five measurands were then extracted from the 3D optical reconstructions using a dedicated image processing software (MountainsMap^®^, Digital Surf, Besançon, France). In detail, the processing was carried out as follows:

- ODt: this diameter was extrapolated from a top acquisition by fitting a circle to the desired points (see Figure 7b).
- *L*_mark_: this quantity was measured as the vertical distance between the highest point belonging to the gate mark and the flat surface from which it protruded (see Figure 7c). This surface was identified in the same way for all the moulded parts by considering a constant height with respect to the plane on which the circle with the ODt diameter lay.
- IDb: this diameter was extrapolated from a bottom acquisition by fitting a circle to the desired diameter points (see Figure 8b). The 3D acquisition was initially processed by applying a levelling and a threshold along the Z-axis. In particular, the threshold allowed to accurately identify only the points belonging to the flash surface, thus eliminating the undesired influence of points acquired inside the hole.
- ODb: this diameter was extrapolated in the same way as IDb. As illustrated in Figure 8b, the outer perimeter of the flash was not as circular as the ones identifying ODt and IDb, making the ODb measurement less reliable than the other ones. This was most probably caused by an imperfect positioning of the central pin inside the mould cavity that created an unbalance in the polymer melt flow inside the micro channel.
- *A*_flash_: the flash area was measured starting from a bottom acquisition by counting the pixels of the flash surface. After the number of pixels, *N*_p_, was determined, a simple equation was used:
  *A*_flash_ = *N*_p_ × *A*_p_ (1)
  where *A*_p_ is the area of one pixel, equal to 0.442 µm^2^. Even though both ODb and *A*_flash_ are indicators of the size of the flash affecting the bottom of the part, the second one is more representative since it is not affected by any circularity error and therefore is more sensitive to any variation of the defect size.

For all moulded parts, each acquisition was repeated three times, and the average of each extracted measurand was taken as output in order to minimize the influence of the instrument repeatability.

The mould geometries corresponding to the three diameters were also measured to provide a reference for calculating the replication capability of the µIM process. In fact, when evaluating replication technologies, especially in the micro-scale and nano-scale where machining accuracy becomes a very challenging task, the knowledge of the master dimensions is of paramount importance to assess the process capabilities [23]. The mould diameters corresponding to ODb and IDb were measured with the aforementioned focus variation microscope by acquiring the hole on the insert and the pin. As for the mould feature correspondent to ODt, no direct measurement procedure was applicable due to the inaccessibility of such a feature for any optical or contact instrument. A fast replication media was therefore used to replicate the internal geometry of the insert. In particular, a brown polyvinylsiloxane (PVS) replication media (AccuTrans^®^, Coltene, Cuyahoga Falls, OH, USA) was casted inside the cavity and measured after solidification. Such a method has been demonstrated to be successfully applicable to indirect measurement tasks where single-digit micrometric accuracy is required [37]. By knowing the mould dimensions, the replication capability Δ*_D_* was defined as follows:

Δ*_D_* = *D*_part_ − *D*_mould_ (2)

where *D*_part_ and *D*_mould_ represent a generic diameter, *D*, measured on the part and the mould respectively. Δ*_D_* is an indicator of the level of accuracy of the µIM process, and therefore the three variables Δ_ODb_, Δ_IDb_ and Δ_ODt_ were considered as responses for the experimental analysis in place of ODb, IDb and ODt.

The quality of the dimensional measurements was verified by calculating the measurement uncertainty, *U*. Such parameter is used to characterize the dispersion of the values that could be reasonably attributed to the measurand [38] and assumes more relevance when the quality of micro products are evaluated. In fact, the uncertainty-to-tolerance ratio, *U/T*, becomes much larger when higher precision levels, typical of the micro-scale, are demanded [10]. Moreover, the measurement uncertainty could, if large enough, partially or totally hide the effects of the experimental variables on the measured output [24]. In this study, the uncertainty related to the five measurands was calculated following ISO 15530-3 [39], which is based on the use of a calibrated artefact sharing similar characteristics with the actual measurand. Two different artefacts were used in the investigation: a calibrated circle of nominal diameter equal to 250 µm for the uncertainty of ODb, IDb and ODt and a calibrated step height of 1 mm for the uncertainty of *L*_mark_. Four uncertainty contributions were considered for the calculation of *U*: *u*_cal_, equal to the one stated in the calibration certificate of the artefact; *u*_res_, due to the resolution of the measurement instrument and calculated by considering a rectangular distribution; *u*_w_, related to the material and manufacturing variations of the actual measurand; and *u*_p_, introduced by the measurement procedure and calculated as a standard deviation of 20 repeated measurements on the calibrated artefact. In particular, *u*_w_ was calculated as:

(3) $$u_{w}\;=\;\frac{\max\left(M\right)-\;\min(M)}{2\sqrt{3}}$$

where **M** is the vector containing the three measurement repetitions of a generic one of the five measurands. The expanded uncertainty, *U*, was finally obtained by combining the contributions:

*U* = *k* × (*u*^2^_cal_ + *u*^2^_res_ + *u*^2^_w_ + *u*^2^_p_)^1/2^ (4)

where the coverage factor *k* was equal to 2 in order to achieve an approximate confidence interval of 95%. As for *A*_flash_, the contributions *u*_cal_ and *u*_p_ were not considered since no calibrated artefact for area measurement was available. Table 5 reports the uncertainty budget for the five measurands involved in the study. It can be seen that for the three diameters, the uncertainty-to-tolerance ratio, *U/T*, ranged between 11% and 12%; such values are satisfying with respect to the upper recommended limit of 20% [26], especially considering the very narrow tolerance range imposed by the design specifications.

### 2.5. Process Monitoring

During each moulding cycle, two process variables were recorded in-line: the pressure, *p*, and the velocity, *v*, of the injection plunger. The two were derived from machine data, and therefore no external sensor was used. Such a type of data is easy to access and available to any machine user, making this analysis particularly interesting for industrial productions, when usually no external sensor is mounted within the moulding machine. In particular, the injection pressure was acquired by means of a strain gauge transducer (Sensorplatte microline, X Sensors AG, Diessenhofen, Switzerland) embedded in the machine and mounted on the back of the injection plunger. The speed of the injection plunger was recorded via the speed of the motor driving the plunger through the control unit of the machine. For both the monitored curves, the frequency of acquisition was set at 167 Hz (equivalent to a sampling interval of 6 ms), corresponding to the maximum value allowed by the machine computer. Pressure and velocity were acquired synchronously by the machine, and therefore the recorded data needed no alignment with respect to the time-scale.

**Figure 9:**
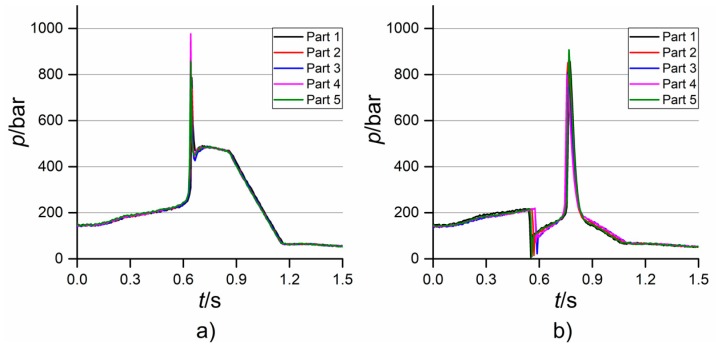
Injection pressure profiles recorded when moulding at: (**a**) high holding pressure and (**b**) low holding pressure. *v*_inj_ was equal to 150 mm/s in both cases.

**Figure 10:**
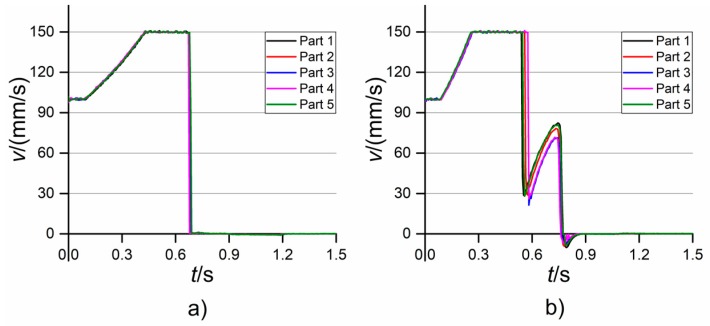
Injection velocity profiles recorded when moulding at: (**a**) high holding pressure and (**b**) low holding pressure. *v*_inj_ was equal to 150 mm/s in both cases.

From the first analysis of the acquired profiles, it was clear that the moulding parameter that mostly affected the shape of the *p* and *v* curves was the holding pressure. In fact, when the high level of *p*_hold_ was selected, a monotone increase of injection pressure was always observed (see Figure 9a). The increase culminated in a peak caused by the high pressure drop due to the extremely small dimensions of the gate and the mould cavity. After the switch-over point, which was defined on the machine by assigning a threshold value of injection pressure, *p* readapted to the negative linear profile of the holding pressure that was set through the machine interface. This profile was chosen since it guaranteed a smooth displacement of the injection plunger. Conversely, when the low value of *p*_hold_ was selected, the injection pressure always showed a point of discontinuity in its profile (see Figure 9b). This behaviour was caused by the plunger suddenly decelerating and then accelerating again. This was because, when moulding with *p*_hold_ equal to 250 bar, the cavity was not filled at the defined switch-over point, thus causing a pressure decrease. After this, the plunger accelerated again during the holding phase, completed the filling, as witnessed by the presence of the pressure peak, and readapted to the imposed negative holding pressure profile. The different level of *p*_hold_ influenced the velocity profiles as well. When moulding with a holding pressure of 500 bar, the plunger accelerated until *v* reached the value of the selected *v*_inj_ and then stopped in correspondence with the switch-over point (see Figure 10a). On the other hand, when moulding with *p*_hold_ equal to 250 bar, *v* suddenly decreased at some point, and then increased again until the final stopping (see Figure 10b). This discrepancy was due, as for the case of *p*, to the decrease in pressure and the consequent piston deceleration that occurred when the cavity was not filled at the defined switch-over point.

**Figure 11:**
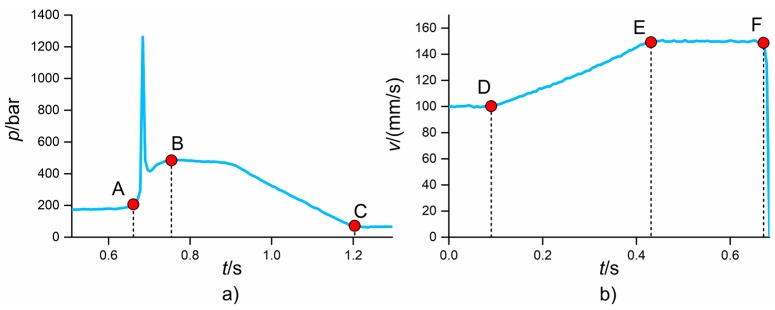
Pressure (**a**) and velocity (**b**) curves with points indicating the time intervals used for calculating the process fingerprints candidates. The position of the points was determined by tracking changes of the slope using the first-order derivative value.

Once *p* and *v* were acquired, their dependence on µIM parameters was studied by identifying some indicators that acted as process fingerprint candidates. In this case, variables that well characterized the shape and main features of both the pressure and velocity curves were selected. In particular, the process fingerprint candidates derived from the injection pressure curves were the following:

- *p*_max_: this quantity was defined as the maximum value for each recorded *p* curve. Being the pressure peak due to the small size of the channels, this is an indicator related to the filling behaviour of the cavity.
- *p*_mean_: this value was calculated as the average pressure in the time interval between the start and the end of the moulding cycle (points A and C in Figure 11a). This quantity provides average information on the pressure acting during one moulding cycle.
- I*_p_*: this is the integral of the pressure in the peak region. The peak region was identified as the time interval spanning from the abrupt increase of *p*, correspondent to the start of the filling, and the point where the injection pressure adapted to the holding pressure profile given in input to the machine. Therefore, this quantity is related to the amount of energy provided by the injection plunger during the filling phase and is expected to be very process-dependent since the filling of the cavity is usually highly influenced by variations of the µIM process parameters. In particular, I*_p_* was calculated by applying the trapezoidal rule:
  (5) $$I_{p}=dt\times \overset{t_{B}-dt}{\underset{{t=t}_{A}}{\sum }}\;\frac{p\left(t\right)+p(t+dt)}{2}$$
  where *t* is the time, *t*_A_ and *t*_B_ are the times correspondent to points A and B respectively (see Figure 11a), and d*t* is the sampling interval of 6 ms correspondent to the sampling frequency of 167 Hz.
- I*_p_*/Δ*t*: this quantity is equal to the integral mean of *p* in the peak region, i.e., I*_p_* divided by the integration range Δ*t* = *t*_B_ − *t*_A_ (see Figure 11a). Such a variable differs from I*_p_* since it is not influenced by the range of integration, i.e., the duration of the filling phase. I*_p_*/Δ*t* is thus an indicator of the average *p* acting during the pressure peak.

From the injection velocity curves, two indicators were extracted:

- *v*_mean_: the mean velocity calculated as the average of the *v* values in the time interval between the start of the acceleration of the plunger and its stop at the switch-over point (point D to point F in Figure 11b), i.e., when *v* starts decreasing towards a null velocity. This quantity is therefore related to the average velocity that characterized the filling phase of the moulding cycle.
- *v*_slope_: the slope of the velocity curve between the start and the end of the acceleration (point D to point E in Figure 11b). This value is equal to the constant acceleration assumed by the plunger to reach the selected *v*_inj_ value.

The maximum value of *v* was not taken into account in the investigation since it was equal to the selected *v*_inj_ and therefore only dependent on that µIM parameter.

## 3. Results and Discussion

### 3.1. Product Fingerprint Analysis

**Figure 12:**
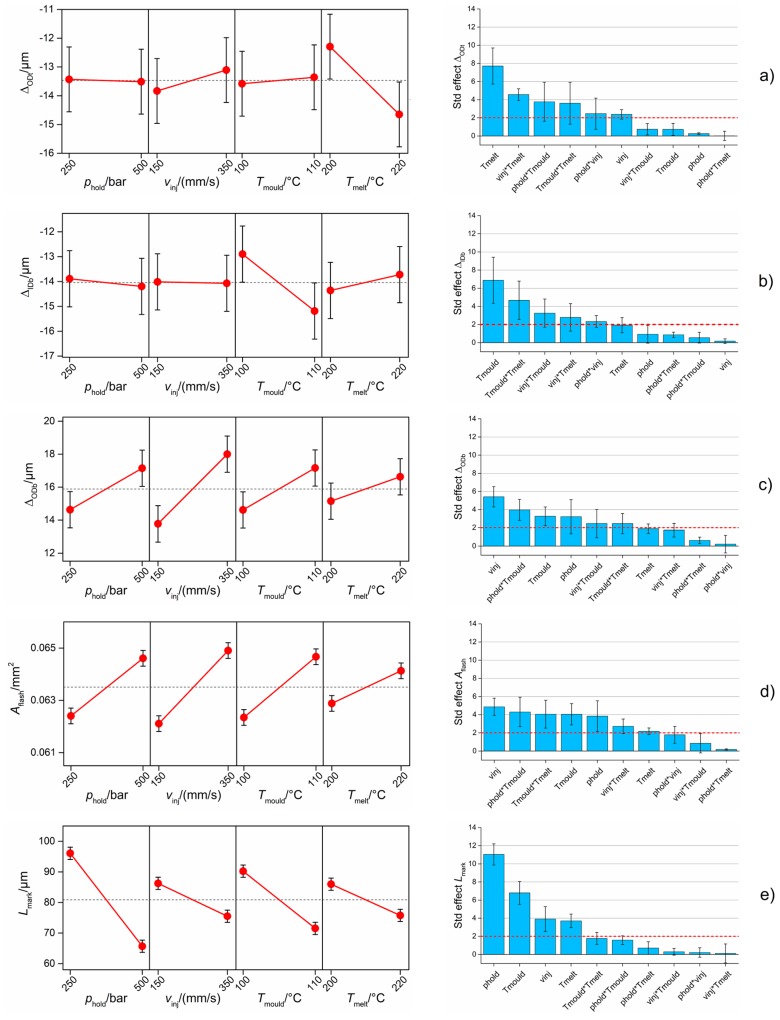
Influence of the µIM process on the five product fingerprint candidates: (**a**) Δ_ODt_, (**b**) Δ_IDb_, (**c**) Δ_ODb_, (**d**) *A*_flash_ and (**e**) *L*_mark_. Main effects plot (left column) and Pareto chart of standardized effects (right column) are reported. The interval bars represent the expanded measurement uncertainties, *U*, in the main effects plots and the standard deviations of five Pareto analyses for the five DoE replicates in the Pareto charts. The red dashed line in the Pareto chart is the significance level at 95% of confidence level.

Figure 12 reports the results of the analysis for the five product fingerprint candidates. The main effects plot and the Pareto chart of the effects are shown: these two graphs allow for the determination of which process parameter had a significant influence on the response in a robust statistical way. In both the plots, interval bars are shown. In the main effects plots, they represent the expanded measurement uncertainty (see Table 5) that must be taken into account when evaluating the effects of process variation on a measurand. In fact, the variability of the process could be entirely covered by the measurement uncertainty, especially for micro manufacturing processes where the induced dimensional variations are typically in the micrometre range [23]. In the Pareto charts, the interval bars represent the standard deviation of the effects obtained by running five separate analyses for the five DoE replicates. The evaluation of such a variability is important since it quantifies the repeatability of the conclusions drawn from the Pareto chart; a low standard deviation of an effect leads to the conclusion that the significance of that effect is robust with respect to process repeatability. In particular, an effect whose interval bar overlaps with the significance limit cannot be considered as significant.

Figure 12a shows the results for Δ_ODt_. For this output, the most significant process parameter was *T*_melt_; its increase led to a decrease in replication fidelity by 2.5 µm. Considering the measurement uncertainty, the other three µIM process parameters did not have a relevant influence on the measured output. As for second-order interactions, only the one between *T*_melt_ and *v*_inj_ had a relevant impact on Δ_ODt_, given that its standardized effect was larger than the significance limit and its standard deviation bar was not overlapping with it.

Figure 12b reports the results for the replication of IDb. On average, the replicated IDb was 14.0 µm smaller than the master. This shrinkage level was very similar to that of ODt. The mould temperature was the only significant process parameter. In particular, increasing *T*_mould_ from 100 °C to 110 °C led to a decrease in replication level. Such a behaviour, which may seem opposite to the usual enhancement of replication obtained when increasing mould temperature, was already observed in the literature when internal features, i.e., pins, were replicated [23]. The Pareto chart of the effects revealed that only the second-order interaction between *T*_mould_ and *T*_melt_ was significant.

Δ_ODb_ was more sensitive to process variations than the previous measurands (see Figure 12c). In particular, variations in *p*_hold_, *v*_inj_, and *T*_mould_ led to an increase in the output that was bigger than the measurement uncertainty. In this case, the results assumed positive values since ODb is the diameter of the circle that identifies the perimeter of the flash formed at the end of the flow (see Figure 8); the replicated diameter was larger than the correspondent one of the micro cavity. Increasing any of the investigated process parameters had a positive effect on Δ_ODb_ and consequently on the flash size. The reason for this is to be found in polymer rheology. When using the high mould temperature, the viscosity of the polymer melt inside the cavity was reduced, thus providing a better replication of the cavity features and, in this case, a bigger flash. The same effect was obtained when increasing the injection speed; the viscosity of the melt was reduced thanks to the shear thinning behaviour of thermoplastic polymers such as POM, thus achieving a longer flow path inside the venting channel that resulted in a larger flash and consequently a larger ODb. Finally, increasing the holding pressure allowed more material to enter the cavity, thus increasing the flash size. These findings were also confirmed by the Pareto chart, which also showed that the second-order interaction between *T*_mould_ and *p*_hold_ had a significant impact on the measured response. Similar effects of the µIM process parameters on the flash size were also reported in [40].

The area of the flash, *A*_flash_, showed the same trends as Δ_ODb_ (see Figure 12d). This was expected since the two dimensional outputs both have a direct relationship with the flash size. However, the measurement uncertainty was less influent in this case. Moreover, according to the Pareto chart, *p*_hold_ can be considered more significant than in the case Δ_ODb_ since the standard deviation of its standardized effect does not overlap with the significance limit. Differently from the previous case, the second-order interaction between the mould and the melt temperature was also influent. Therefore, it can be concluded that *A*_flash_ was more sensitive to process variations than Δ_ODb_, thus representing a more suitable product fingerprint candidate based on the flash size.

**Figure 13:**
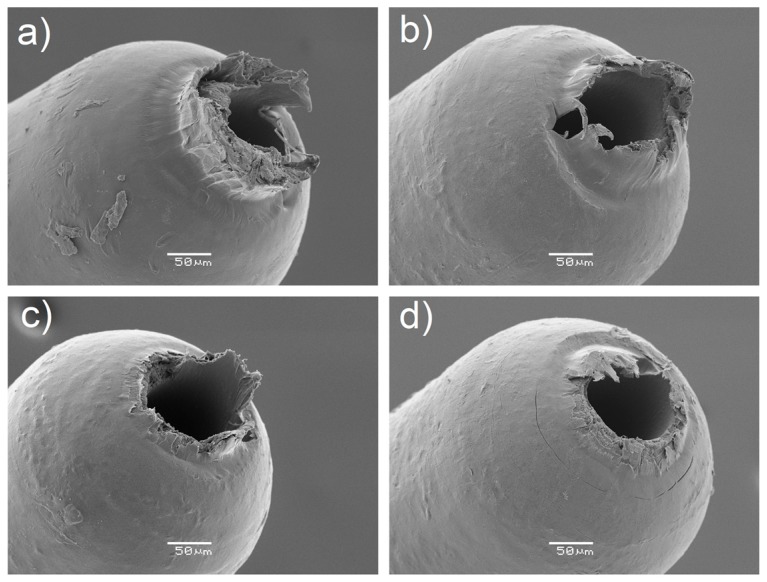
Gate mark appearance for different combinations of process parameters: (**a**) was moulded with low levels of *T*_melt_, *p*_hold_, *T*_mould*,*_ and *v*_inj_; (**b**) was moulded with low levels of *p*_hold_, *T*_mould_*,* and *v*_inj_; (**c**) was moulded with low levels of *p*_hold_; and (**d**) was moulded with high levels of the four process parameters.

Figure 12e shows the results for the gate mark length, *L*_mark_. The size of this defect was on average equal to 82 µm, which is relevant if compared to the overall component dimensions (see Figure 1). All four investigated process parameters had a significant influence on *L*_mark_ according to the main effects plot and Pareto chart. In particular, an increase in the variables led to a decrease in the defect size, demonstrating that such a product fingerprint was very sensitive to µIM settings variations. The holding pressure showed the largest impact, since the use of 500 bar provided parts having on average a 30 µm shorter gate mark than those moulded with *p*_hold_ equal to 250 bar. In order to further inspect the effects of µIM on this defect, SEM images of parts manufactured with different DoE combinations were taken and compared (see Figure 13). It can be observed that when moulding with the low levels of the four parameters, the gate mark was very evident, almost occluding the upper hole of the component and thus disabling its functionality. A zone of deformation was clearly visible around the defect, meaning that the mechanism that generated the defect was a ductile breakage caused by the detachment of the gate from the part. Setting the µIM parameters at a high level allowed a great reduction of the gate mark size (see Figure 13b,c) and of the area of deformation. Finally, selecting the high levels of all the four process variables generated parts almost free of the defect. The great influence of the process conditions on the gate mark size and appearance may have been caused by a change in the crystallinity of the polymer. In particular, moulding with high levels of the process parameters may have impeded the formation of crystals because of the more drastic cooling rate, thus decreasing the mechanical properties and in turn facilitating the brittle detachment of the gate from the part. It is worth noting that *p*_hold_, *v*_inj_, *T*_mould_ and *T*_melt_ had an opposite effect on the size of *A*_flash_ and *L*_mark_, i.e., the two defects affecting the part quality. This means that a simultaneous minimization of the two defects was impossible inside the investigated experimental range.

The analysis of the effects of the µIM parameters on the five product fingerprint candidates allowed some conclusions to be made. In particular, the best candidates with respect to the characteristic of process sensitivity were *A*_flash_ and *L*_mark_; both were greatly influenced by all the four investigated parameters and can therefore serve to tune the process with the aim of optimizing the quality of the produced parts.

Along with the sensitivity to process variation, the other characteristic that an effective product fingerprint must have is the correlation to the overall part quality. This is necessary to guarantee an efficient quality control based only on the measurement of a single fingerprint. In order to identify the best fingerprint candidate with respect to this requirement, a correlation analysis was carried out. In particular, the coefficient of correlation, ρ, was calculated for each couple of measurands. ρ was calculated as follows:

(6) $$\rho \left(x,\;y\right)=\frac{\sum (x\;-\;\bar{x}\;)(y\;-\;\bar{y}\;)}{\sqrt{\sum {\left(x\;-\;\bar{x}\;\right)}^{2}\sum {\left(y\;-\;\bar{y}\;\right)}^{2}}}$$

where **x** and **y** are generic vectors containing two datasets and $\bar{x}$ and $\bar{y}$ are their respective mean values. Such a coefficient can vary between −1 and +1, where the first describes a perfect negative correlation and the second a perfect positive correlation. A ρ equal to 0 indicates that no correlation exists between the two examined sets of values. In this study, ρ was calculated by considering all the 80 values derived from the DoE campaign for each of the five measurands.

**Figure 14:**
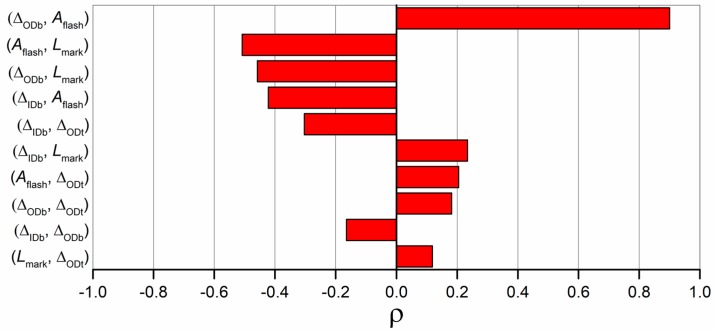
Values of the coefficients of correlation calculated between each couple of measured datasets. Values were sorted largest to smallest in absolute value.

Figure 14 shows the correlation coefficients calculated for the ten couples of product fingerprint candidates. It can be seen that the greatest correlation (ρ = 0.9) was the one between Δ_ODb_ and *A*_flash_. The two measurands are in fact strictly related to the flash size, as explained before. Therefore, an increase in one determined an increase in the other and vice versa, meaning that controlling only one of them allowed for the monitoring of both the geometrical outputs. A second group of coefficients of correlation ranging from −0.5 to −0.4 can be identified. *A*_flash_ and *L*_mark_ shared in fact a ρ equal to −0.5, demonstrating that a significant amount of correlation existed between these two product fingerprint candidates. This was also mirrored in the main effects plots (see Figure 12) where it was clear how the four investigated process parameters had an opposite effect on the two defect sizes. Significant negative correlations were also observed between Δ_ODb_ and *L*_mark_ and between Δ_IDb_ and *A*_flash_. The second one was due to the geometry of the flash; a decrease in IDb resulted in an increase in the flash area according to its definition (see Figure 8). The other coefficients of correlation were all lower than 0.3 in absolute value and thus negligible if compared to the others.

From this analysis, it can be concluded that the product fingerprint candidate that was the most correlated to the overall part quality was *A*_flash_ since its values were the ones that showed high levels of ρ in combination with three other measurands, namely Δ_ODb_, *L*_mark_, and Δ_IDb_. Therefore, by controlling only this dimensional characteristic of the moulded part, the best control over the overall part quality can be carried out. Considering also the requirement of sensitivity to process settings variations, the best product fingerprint for the specific part under analysis was the flash area, followed by the length of the gate mark. Such quality indicators must be related to in-line, monitored process variables in order to function in a fast and comprehensive µIM assurance strategy.

### 3.2. Process Fingerprint Analysis

A similar type of analysis was carried out to identify the best process fingerprint candidate among the six indicators derived from the monitored pressure and velocity curves with respect to sensitivity to process variations. In this case, the main effects plots were represented with interval bars equal to the standard errors calculated among the values related to the particular combination of process parameters.

**Figure 15:**
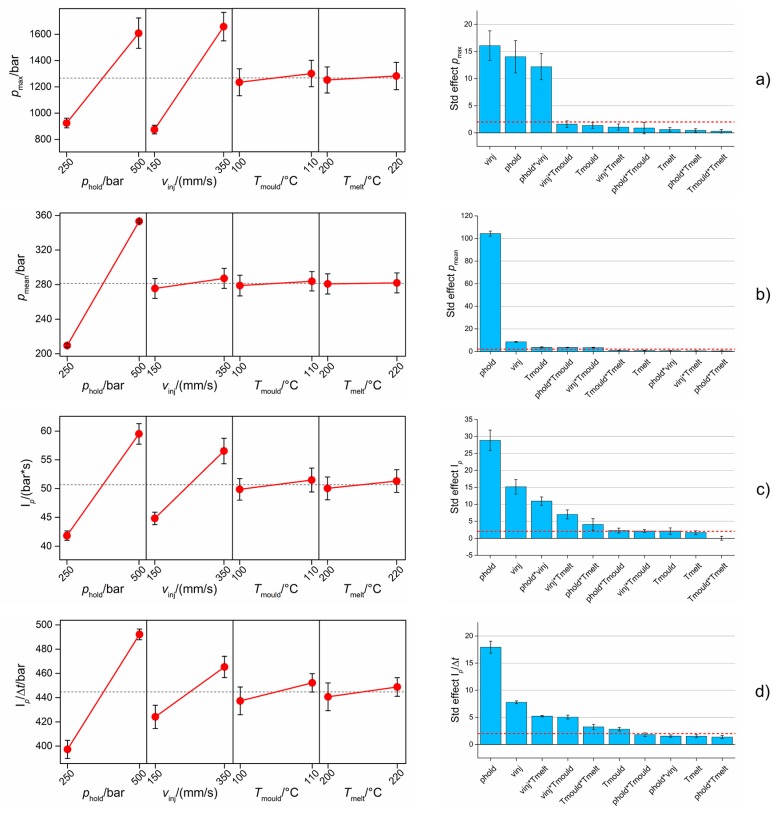
Influence of µIM process on the four process fingerprint candidates derived from monitored injection pressure curves: (**a**) *p*_max_, (**b**) *p*_mean_, (**c**) I*_p_* and (**d**) I*_p_*/Δ*t*. Main effects plot (left column) and Pareto chart of standardized effects (right column) are reported. The interval bars represent the standard errors in the main effects plots and the standard deviations of five Pareto analyses for the five DoE replicates in the Pareto charts. The red dashed line in the Pareto chart is the significance level at 95% of confidence level.

Figure 15a shows the results for *p*_max_. This indicator depended mainly on the selected *p*_hold_ and *v*_inj_ values. Particularly, an increase in both the holding pressure and the injection speed resulted in an increase of the maximum injection pressure. The effect of *v*_inj_ can be explained by considering that flowing a fluid with a higher speed requires a higher pressure. The effect of *p*_hold_ is, on the other hand, due to the used modality of switch-over; as already anticipated, the machine was set to switch from the filling phase to the holding one when a certain pressure was reached. Therefore, when selecting a higher *p*_hold_, the injection pressure was allowed to rise more before switching to the holding profile. The second-order interaction between *p*_hold_ and *v*_inj_ was also significant.

*p*_mean_ was predominantly influenced by the holding pressure (see Figure 15b). This was caused by the fact that *p*_mean_ was calculated as the average *p* among the entire moulding cycle (see Figure 11a). The averaging operation, in fact, minimized the relevance of the peak phase, making the holding phase preponderant, and thus *p*_hold_ became the most significant term. All the other effects, second-order interactions included, were negligible with respect to the holding pressure significance.

Figure 15c illustrates the effect of µIM process parameters on I*_p_*. This fingerprint candidate was mainly influenced by holding pressure. The second most significant effect was that of *v*_inj_. The reason for this is similar to the one explained when commenting on *p*_max_ dependence on the process variations. In fact, the integral of *p* in the peak region was strongly influenced by the *p* peak. However, in this case, there was a larger sensitivity with respect to other process parameters such as *T*_mould_, which had a larger impact on the results. However, considering the interval bars of both the main effects plot and the Pareto chart, the mould temperature cannot be considered as significant for I*_p_*.

I*_p_*/Δ*t* showed a process dependence very similar to I*_p_* (see Figure 15d). However, the operation of normalizing the pressure integral over the integration range considerably diminished the relevance of *v*_inj_ while enhancing that of *T*_mould_, which was significant according to the Pareto chart even though the interval bars of the main effects plot slightly overlapped. This may have been caused by the fact that changing the injection velocity setting had an impact on the duration of the peak region and therefore on the filling time. In particular, increasing the mould temperature resulted in an increase in I*_p_*/Δ*t*. This is somehow unexpected since increasing *T*_mould_ usually decreases the polymer viscosity and therefore the pressure needed to drive the melt through the cavity channels. As for the previous case, second-order interactions of *v*_inj_ had a significant effect on this output.

**Figure 16:**
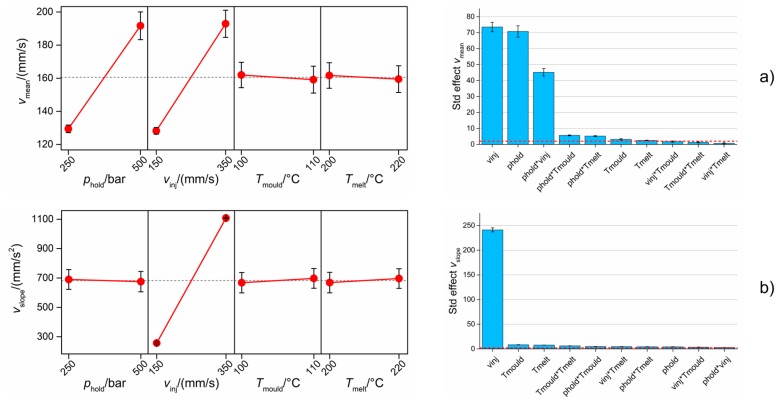
Influence of µIM process on the two process fingerprint candidates derived from monitored injection velocity curves: (**a**) *v*_mean_ and (**b**) *v*_slope_. Main effects plot (left column) and Pareto chart of standardized effects (right column) are reported. The interval bars represent the standard errors in the main effects plots and the standard deviations of five Pareto analyses for the five DoE replicates in the Pareto charts. The red dashed line in the Pareto chart is the significance level at 95% of confidence level.

Figure 16a shows the results for *v*_mean_, whose value was influenced mostly by *v*_inj_, *p*_hold_ and their interaction. Increasing the injection speed had a positive effect on *v*_mean_, since a higher velocity plateau was recorded. Increasing the holding pressure had the same effect; this was caused by the fact that when moulding at the high level of *p*_hold_, no deceleration of the plunger, and therefore no decrease in speed during the filling phase, was observed (see Figure 10b). The significance of the interaction was caused by the fact that when using high *p*_hold_, an increase in injection speed led to a larger increase in *v*_mean_ than when using a low *p*_hold_. In fact, the deceleration behaviour was similar when using both high and low *v*_inj_.

*v*_slope_ depended mainly on the value of the injection speed (see Figure 16b). In particular, moulding at a high *v*_inj_ determined a substantial increase in the plunger acceleration from 260 mm/s^2^ to 1100 mm/s^2^. This means that when a higher *v*_inj_ was selected, the machine motor provided the plunger with a higher acceleration in order to reach it.

From this analysis, it can be concluded that holding pressure and injection speed were the most influencing parameters for the six process fingerprint candidates derived from monitored injection pressure and velocity curves. On the other hand, mould and melt temperature variations were in most cases not significant in determining the level of the responses. This was because pressure and velocity were both measured at the injection plunger location. Quantities measured inside the mould with external sensors proved in fact to be more sensitive to *T*_melt_ and *T*_mould_ variations [18]. Only the integral mean of the pressure, I*_p_*/Δ*t*, showed a relevant dependence on *T*_mould_, making this indicator the best process fingerprint candidate among those extracted from the *p* and *v* curves with respect to the sensitivity to µIM process variations. In fact, by in-line monitoring I*_p_*/Δ*t*, variations of holding pressure, injection speed and mould temperature can be observed and quantified.

In order to investigate the relation between I*_p_*/Δ*t* and the part quality, which is a fundamental characteristic of a process fingerprint to allow an effective in-line quality monitoring, the same approach used for the product fingerprint correlation analysis was adopted. In particular, the coefficient of correlation, ρ, was calculated using Equation (6) among I*_p_*/Δ*t* and the five dimensional measurands.

**Figure 17:**
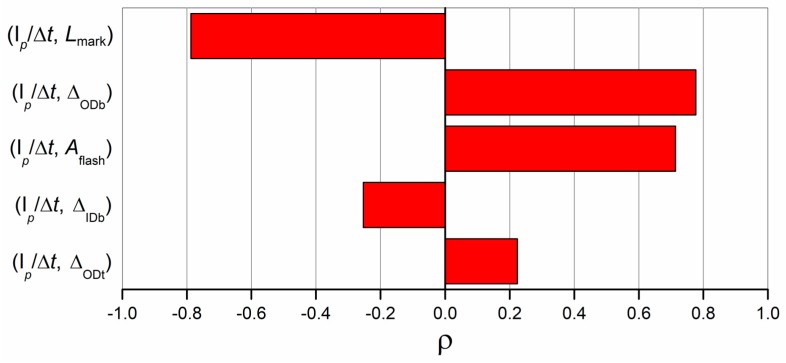
Values of the coefficients of correlation calculated between I*_p_*/Δ*t* and the five dimensional measurands. Values were sorted largest to smallest in absolute value.

Figure 17 shows the results. The correlation coefficient values appear as divided in two distinct subgroups: one made of ρ values calculated for *L*_mark_, Δ_ODb_ and *A*_flash_, which were all larger than 0.7 in absolute value, and the other made of ρ values calculated for Δ_IDb_ and Δ_ODt_. This means that I*_p_*/Δ*t* was highly correlated with the size of the gate mark and the flash, i.e., the defects affecting part quality, and with the dimension of ODb, which was in turn highly related to *L*_mark_ and *A*_flash_ (see Figure 14). On the other hand, the other two measurands showed no relevant link with the integral mean of *p*, being the correlation coefficients values equal to −0.25 and 0.21 for Δ_IDb_ and Δ_ODt_ respectively. These findings proved that I*_p_*/Δ*t*, besides being sensitive to variations in µIM process parameters, was also correlated to the two best product fingerprint candidates, namely the flash area and the length of the gate mark. Therefore, it can act as the bridge between process monitoring and part quality, representing the link needed to perform a faster and more comprehensive assurance of the investigated moulded component.

**Figure 18:**
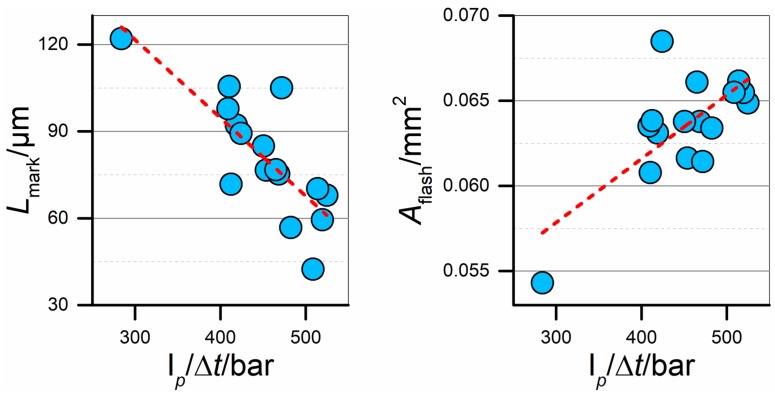
Correlation plots of the two defect size indicators against I*_p_*/Δ*t*. Red dashed lines represent linear trends. Each point represents the average of the five DoE replicates.

Figure 18 shows the plots of gate mark and flash sizes against I*_p_*/Δ*t*. It can be observed that there indeed existed a relation among the data. In particular, there is an almost linear correlation between *L*_mark_ and the integral mean. The data also show a limited dispersion around the depicted linear trend, demonstrating that the relation between the two variables was robust inside the investigated experimental range. Since the trend is negative, a higher I*_p_*/Δ*t* determined a smaller gate mark on the moulded parts. This finding agrees with the opposite slopes of the main effects plots for the two variables (see Figure 12e and Figure 15d). By controlling the process fingerprint, an accurate control on the size of this defect can be performed: I*_p_*/Δ*t* must be kept at values around 500 bar if the size of the gate mark has to be minimized. Conversely, I*_p_*/Δ*t* equal to 300 bar generated a defect having double the size. In regards to the size of the flash, indicated by the *A*_flash_ value, a positive relation was observed. Therefore, lower I*_p_*/Δ*t* values were needed to minimize the size of this defect; the lower the pressure applied during filling, the smaller the flash on the component. As already anticipated, the minimization of both defects was not possible within the investigated process parameters ranges. In this case, the data were more dispersed than for *L*_mark_, resulting in a less precise and accurate prediction. However, the general data trend is still evident, as shown by the high coefficient of correlation of 0.71 (see Figure 17). As *A*_flash_ correlated to most of the other dimensional measurands (see Figure 14), the optimization of the part quality can be effectively carried out by monitoring I*_p_*/Δ*t* for every moulding cycle, thus assessing the quality of all moulded parts.

## 4. Conclusions

The present paper aimed at applying and validating a new optimization concept based on the product and process fingerprints of the µIM process of a micro medical component. The best product and process fingerprint candidates were identified by considering two characteristics: sensitivity to process variations and correlation to part quality. Optical metrology and process monitoring using no external sensors were, for the first time in reported literature, successfully combined to provide an in-line optimization strategy in µIM.

The following conclusions are drawn from the study:

- The variation of µIM process settings had a relevant impact on the quality of the micro component. In particular, the flash area and the length of the gate mark showed the largest sensitivity.
- Varying the four investigated process parameters had an opposite effect on the size of the two defects: an increase in the flash size always came with a decrease in the gate mark size and vice versa. Their simultaneous minimization was therefore not possible to obtain within the investigated process window, posing a great challenge with respect to quality optimization.
- The morphology of the gate mark was deeply influenced by the selected process settings. In particular, a zone of deformation was clearly visible only when moulding with low levels of the parameters, thus significantly increasing the size of the defect.
- The flash area was the measurand with the highest level of correlation to part quality. By measuring the effects of µIM parameters on such indicators, robust conclusions can be made also on three other measurands, namely ODb, *L*_mark_ and IDb. Therefore, *A*_flash_ represented the best product fingerprint candidate for the analysed component.
- The indicators extracted from in-line monitored injection and velocity curves were mostly influenced by *p*_hold_ and *v*_inj_. The only one that showed a significant dependence on another parameter, namely *T*_mould_, was the mean integral of the pressure during filling, I*_p_*/Δ*t*. This variable increased when selecting the high levels of the µIM parameters. Being the most sensitive among the investigated process indicators, it was chosen as the best process fingerprint.
- I*_p_*/Δ*t* showed a significant correlation with three measurands. In particular, the size of both the defects could be effectively controlled by monitoring the I*_p_*/Δ*t* value for each moulding cycle. Such a discovery demonstrated that in-line process optimization in µIM can be carried out by means of a robust monitoring strategy in order to make sure that all the manufactured components have dimensions within the desired range.

Future work will be dedicated to the application of a similar approach to different types of moulded samples such as micro structured and nano structured surfaces. This will extend the validity of this approach to other dimensional ranges and classes of components.

## Figures and Tables

**Table 1:** Examples of micro-injection moulding (µIM) components and their volumes.

Micro component	Approximate Volume/mm^3^	Reference
Micro filter	1.0	[11]
Micro ring	2.5	[23]
Part for weld line investigation	6.5	[24]
Part with micro pillars	12.0	[17]
Micro gear	14.0	[25]
Toggle for hearing aids	22.0	[26]
Dog-bone tensile bar	28.0	[27]
Thin-walled part	31.5	[28]
Square part for shrinkage evaluation	35.0	[29]
Cylindrical support with micro pillars	110.0	[30]
Disco with micro features and nano features	113.5	[31]

**Table 2:** Main properties of the polyoxymethylene (POM) grade.

Property	Unit	Value	Test Method
Density	kg/m^3^	1410	ISO 1183
Melt volume rate (*T* of 190 °C, load of 2.16 kg)	cm^3^/10min	24	ISO 1133
Melting temperature	°C	166	ISO 11357-1, -2, -3

**Table 3:** Design of Experiments (DoE) process settings.

Process Parameter	Symbol	Unit	Low Level	High Level
Holding pressure	*p*_hold_	bar	250	500
Injection speed	*v*_inj_	mm/s	150	350
Mould temperature	*T*_mould_	°C	100	110
Melt temperature	*T*_melt_	°C	200	220

**Table 4:** Focus variation microscope characteristics.

Instrument Characteristic	Value
Objective magnification	20×
Numerical aperture	0.40
Working distance/mm	13.0
Field of view/µm	714 × 542
Digital lateral resolution/µm	0.44
Declared vertical resolution/nm	0.14

**Table 5:** Mean values of uncertainty contributions and expanded uncertainty for the five measurands.

Uncertainty Contribution	ODt/µm	IDb/µm	ODb/µm	*L*_mark_/µm	*A*_flash_/µm^2^
*u*_cal_	0.50	0.50	0.50	0.45	/
*u*_res_	0.13	0.13	0.13	0.04	0.19
*u*_w_	0.19	0.20	0.16	0.73	1.5 × 10^2^
*u*_p_	0.12	0.12	0.12	0.37	/
*U* (*k* = 2)	1.1	1.1	1.1	1.9	3.0 × 10^2^
